# Early selection of novel triploid hybrids of shrub willow with improved biomass yield relative to diploids

**DOI:** 10.1186/1471-2229-14-74

**Published:** 2014-03-24

**Authors:** Michelle J Serapiglia, Fred E Gouker, Lawrence B Smart

**Affiliations:** 1 Department of Horticulture, Cornell University, New York State Agricultural Experiment Station, Geneva, NY 14456, USA

**Keywords:** Allometrics, Heterosis, Hybrids, Polyploids, Specific gravity, Willow

## Abstract

**Background:**

Genetic improvement of shrub willow (*Salix*), a perennial energy crop common to temperate climates, has led to the development of new cultivars with improved biomass yield, pest and disease resistance, and biomass composition suitable for bioenergy applications. These improvements have largely been associated with species hybridization, yet little is known about the genetic mechanisms responsible for improved yield and performance of certain willow species hybrids.

**Results:**

The top performing genotypes in this study, representing advanced pedigrees compared with those in previous studies, were mostly triploid in nature and outperformed current commercial cultivars. Of the genotypes studied, the diploids had the lowest mean yield of 8.29 oven dry Mg ha^−1^ yr^−1^, while triploids yielded 12.65 Mg ha^−1^ yr^−1^, with the top five producing over 16 Mg ha^−1^ yr^−1^. Triploids had high stem area and height across all three years of growth in addition to greatest specific gravity. The lowest specific gravity was observed among the tetraploid genotypes. Height was the early trait most correlated with and the best predictor of third-year yield.

**Conclusions:**

These results establish a paradigm for future breeding and improvement of *Salix* bioenergy crops based on the development of triploid species hybrids. Stem height and total stem area are effective traits for early prediction of relative yield performance.

## Background

As a fast-growing renewable feedstock, shrub willow has the potential to off-set fossil fuel usage and contribute to the production of renewable fuel products with positive environmental impacts. As the demand for bioenergy and bioproducts continues to rise, shrub willow will be a significant source of woody biomass, because of its fast growth, ease of cultivation, flexible harvesting window, and suitable biomass characteristics for conversion. Shrub willow is an established bioenergy crop in Europe and interest is expanding in the United States and Canada. Current research efforts are focused on improving willow biomass traits important to the renewable fuels industry, including production yield and biomass composition. Breeding and development of shrub willow began in the mid-1980s and the production of new genotypes through intra-specific crosses and inter-specific hybridization has led to the commercialization of many cultivars suitable for bioenergy production [1,2].

Genetic improvement of shrub willow utilizes controlled pollination of this dioecious and highly heterozygous species and then selection of superior genotypes for subsequent clonal propagation. The genus *Salix* consists of approximately 300 species with ploidy levels that range among species from diploid up to dodecaploid and can even vary among individuals within a species [3-5]. Species hybridization leading to heterosis has been exploited by many breeders to improve growth and vigor in several woody species, including willow, although little is known about the genetic basis for this phenomenon [6,7]. Heterosis has long been studied and theories to explain the genetic basis of this phenomenon were first proposed a century ago and are still being refined today [8-10]. Recent studies using advanced genetic and genomic approaches demonstrate that the genetic mechanisms behind heterosis are complex and varied among different systems and may well involve combinations of effects explained by the dominance, overdominance, and epistasis hypotheses [10-13]. Heterosis is commonly observed in polyploid individuals, which is the case for the allopolyploid crops, wheat and cotton. Willow and poplar triploids, in general, have been known to display improved vigor, but the basis for this observation has not been studied [5,14,15].

One objective of this study was to evaluate progeny from crosses performed in 2001, 2002, and 2005, in particular to determine any association of heterosis for growth traits and ploidy. Many of the initial breeding efforts for shrub willow focused on first-generation intra-specific crosses and inter-specific hybridization, utilizing natural accessions of willow throughout the native region. Progeny from initial rounds of breeding have displayed significant gains in biomass yield, many of which have been tested in multiple yield trials and scaled up for commercial production [2,16,17]. Improvements in breeding and selection of shrub willow through the development of more complex pedigrees, compared to those in previous years, has potential to increase yield productions.

As breeding programs advance through multiple rounds, the intensity of selection will likely need to be increased to continue to achieve genetic gain. This can be accomplished through evaluation of larger populations of progeny, but this requires rapid early screening to reduce the costs associated with long-term field trials. In addition to evaluating heterosis, the current trial was used to examine the value of early measurements that could be used to screen large numbers of progeny and predict yield rankings through allometrics. Combined, the objectives of this study will not only help us understand the morphological variance among different pedigrees of shrub willow, but will also provide a framework for designing new strategies for early selection and crop improvement.

## Results

### Breeding to produce novel pedigrees

Willow breeding was performed in 2001, 2002, and 2005 by Richard Kopp and Kimberly Cameron producing over 3,000 progeny. All individuals were planted in single plant, non-replicated Family Screening Trials at LaFayette Road Experiment Station, Syracuse, NY, during the following spring. Each trial was coppiced after the first growing season. After coppice and the three following growing seasons, all plants were measured for survival, total stem area, height, and were monitored for incidence of pest and diseases. The best individuals from these data were scaled-up into a 75-clone selection trial planted in Geneva, NY USA in 2008 to test the performance of progeny relative to parents (Additional file 1). Each genotype was planted in 24-plant plots in a randomized complete block design with three replicate blocks. Commercial cultivars were included within the trial and are indicated by their cultivar epithet and all non-commerical genotypes are identified by their clone ID.

### Ploidy of parental and progeny genotypes

Flow cytometry was used to estimate ploidy levels of 42 of the 75 genotypes in the trial, selected to represent all of the parents and at least one progeny genotype from each family. Genotypes were determined to be diploid, triploid, or tetraploid based on nuclear DNA content relative to well characterized diploid (*S. purpurea* 94006) and tetraploid (*S. miyabeana* ‘SX64’) parental genotypes included in each batch of analysis (Additional file 1). Nuclear DNA content measurements were significantly different among ploidy groupings (p < 0.01), as determined by mean separation test. The majority of genotypes (38) were determined to be diploid based on nuclear DNA content values ranging between 0.77-1.17 pg 2C^−1^ or were inferred to be diploid based on pedigree. These included selected genotypes of three pure species (*S. purpurea*, *S. eriocephala*, and *S. suchowensis*). All of the genotypes classified as *S. miyabeana* (9) were tetraploid according to flow cytometry with values ranging from 1.48-1.73 pg 2C^−1^ or were inferred based on pedigree.

All of the genotypes selected for flow cytometry that were progeny of diploid by tetraploid crosses were triploid based on nuclear DNA content values ranging from 1.15-1.29 pg 2C^−1^. The other progeny in those families were inferred to be triploid, so that among the 75 genotypes in the trial, 26 (34%) were triploid in ten families from six different species hybrid combinations. While we did not conduct flow cytometry on all 75 genotypes, all the parents and at least one progeny genotype from each family were tested and conformed to the predicted ploidy based on parental ploidy (diploid progeny from diploid parents, tetraploid progeny from tetraploid parents, and triploid progeny from diploid crossed with tetraploid). There were three progeny derived from crosses between a diploid mother and a triploid father. One of those (‘Sheridan’) is triploid based on flow cytometry, while the other two from a different pedigree were designated as tetraploids (01X-264-024 and 01X-264-033) based on DNA content values (1.85 and 1.82 pg 2C^−1^, respectively).

### Growth and biomass production

**Figure 1:**
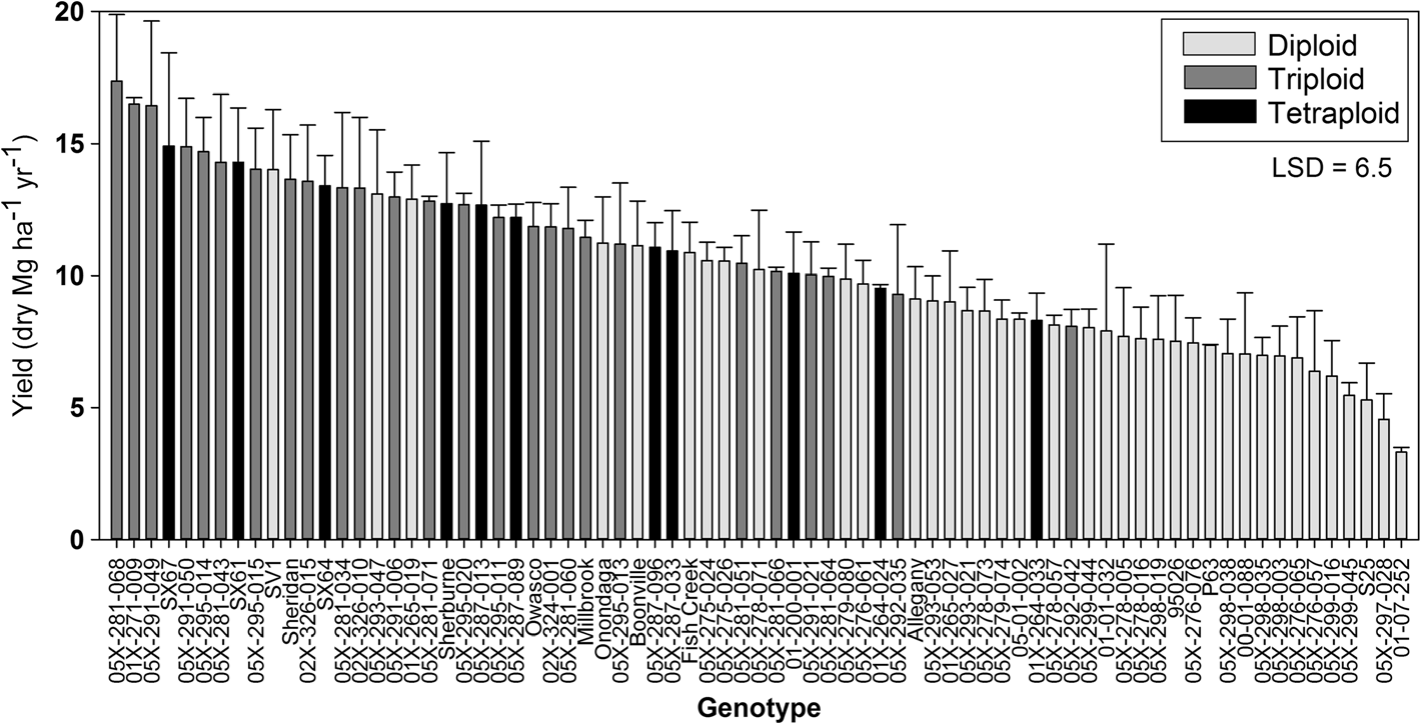
**Harvested biomass per plot at three years post**-**coppice.** Bars represent mean ± standard error. Bars are shaded by ploidy of the genotype.

Of the 75 genotypes harvested in this trial, total stem biomass yield ranged from 2.67 Mg ha^−1^ yr^−1^ to 17.35 Mg ha^−1^ yr^−1^ (Figure 1 and Additional file 2). The highest yielding genotype was a triploid hybrid, (*S. koriyanagi* × *S. purpurea*) × *S. miyabeana* 05X-281-068, and the lowest yielding genotype was a wild, native accession of *S. eriocephala*, 01-07-252. The mean yield of all triploid genotypes was greater than the mean yields of the diploids or tetraploids (p-value <0.0001). Among the top 50^th^ percentile (38 genotypes) for yield, there were 21 triploids, 8 tetraploids, and only 9 diploids, while among the bottom 50^th^ percentile (37), there were 29 diploids, 3 tetraploids, and only 5 triploids. Many of the poor-yielding diploids were *S. eriocephala*, a native species in the US.

**Figure 2:**
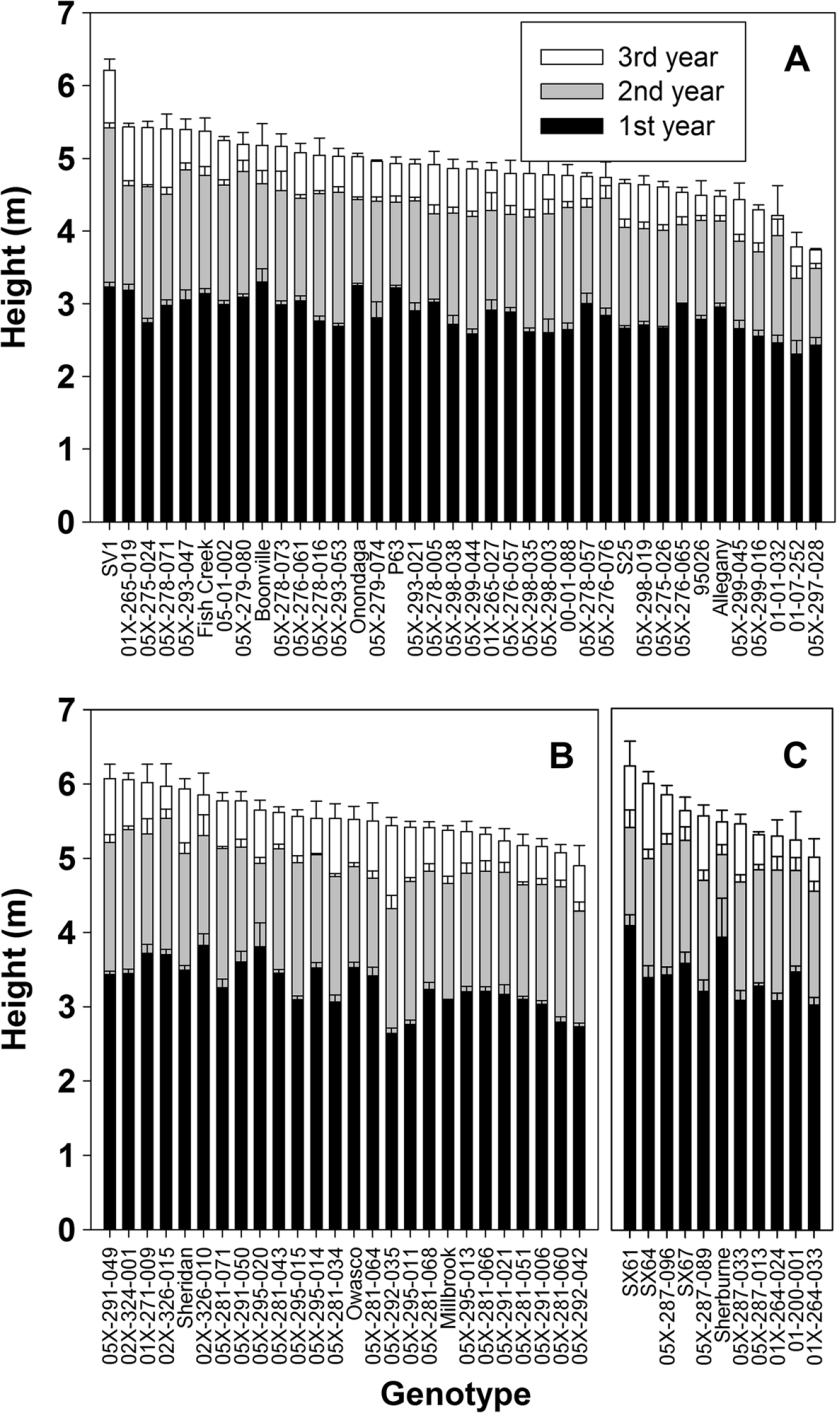
**Average height per plot after each year of growth post**-**coppice.** Bars represent mean ± standard error. **A)** Diploid genotypes, **B)** Triploid genotypes, **C)** Tetraploid genotypes.

**Figure 3:**
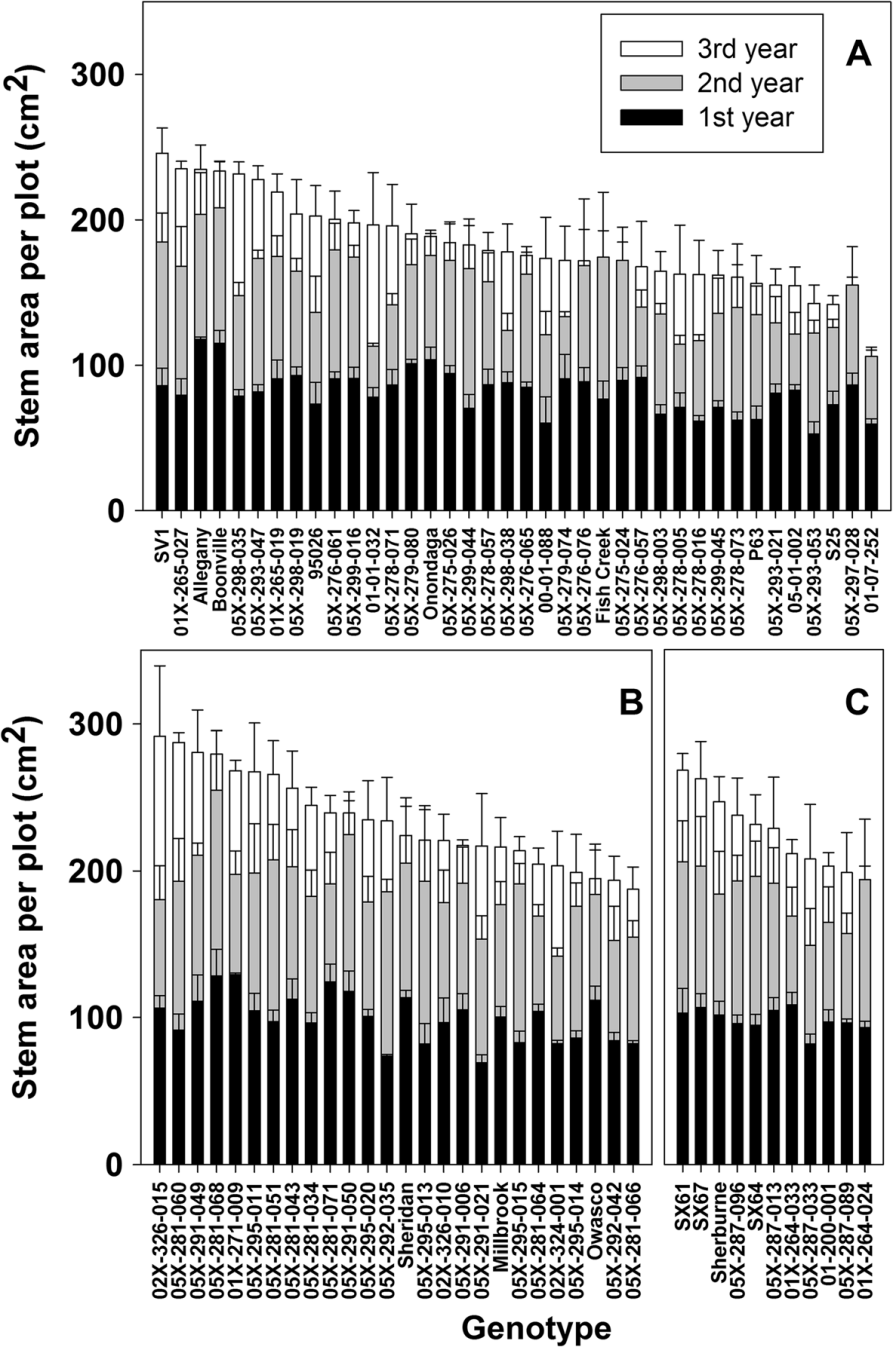
**Total stem area per plot after each year of growth post**-**coppice.** Bars represent mean ± standard error. **A)** Diploid genotypes, **B)** Triploid genotypes, **C)** Tetraploid genotypes.

**Figure 4:**
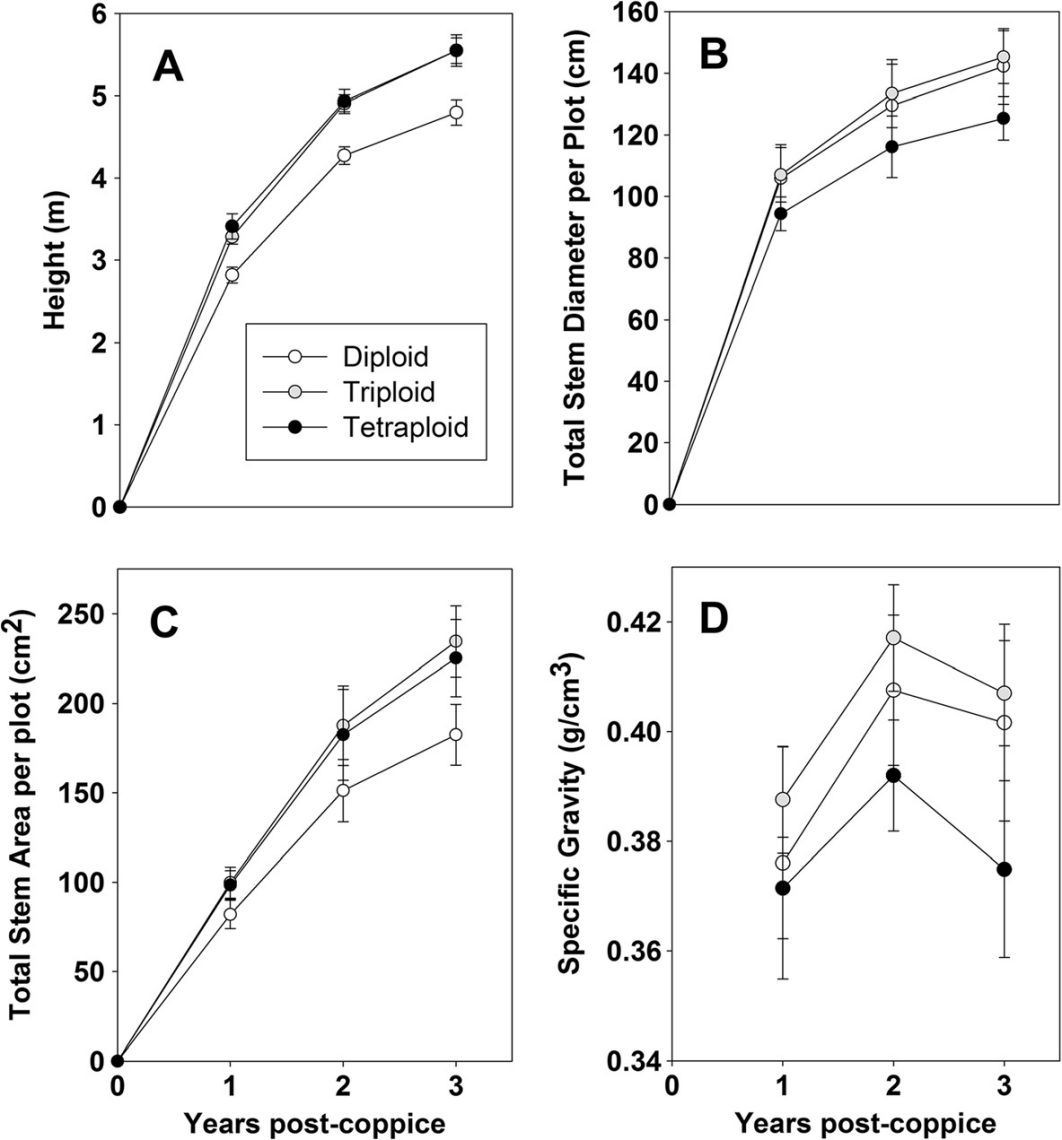
**Growth measurements and specific gravity after each year of growth post**-**coppice averaged by ploidy. A)** Height, **B)** Total stem diameter per plot, **C)** Total stem area per plot, **D)** Specific gravity.

Measurements taken at the end of each growing season included height of the tallest stem per plant, diameter of every stem, stem number per plant, and specific gravity of a stem segment. The stem diameter measurements were used to calculate the mean total stem diameter per plot and mean total stem area per plot. All traits were significantly different by year and genotype (Table 1). Both height and total stem area had a significant interaction by year and by genotype. A significant interaction for total stem area by year and by ploidy was also identified. The greatest height after three years of growth was observed for *S. miyabeana* ‘SX64’, a tetraploid and a parent of many of the triploid progeny (Figure 2). The majority of height growth occurred in the first year post-coppice. The tallest diploid after year three was *S*. × *dasyclados* ‘SV1’, which was significantly taller than any other diploid, with height more similar to the triploids and tetraploids. By ploidy level, mean total stem area was greatest for the triploids, compared with mean values for diploids and tetraploids (Figure 3). There were several diploid genotypes with large total stem area, including ‘SV1’ with the greatest, however the majority of diploids had mean values below 200 cm^2^. Several diploid genotypes lost stem area due to stem dieback from year two to year three, including: *S. purpurea* ‘Fish Creek’, *S. purpurea* × *S. suchowensis* 05X-275-024, (*S. koriyanagi* × *S. purpurea*) × *S. purpurea* 05X-297-028, and *S. purpurea* 01-07-252. Stem number was significantly different by ploidy, with diploids having the greatest mean stem number per plot, 115.4, while the mean for triploids was 101.0, and tetraploids had the lowest mean number of stems per plot, 78.4. While diploids had the greatest number of stems, the total area of those stems was the smallest regardless of year (Figure 4). Diploids had the shortest mean height compared with triploids and tetraploids (Figure 4), but the general growth trend from year to year was very similar regardless of ploidy. Diploids had the greatest total stem diameter, but the smallest total stem area, whereas tetraploids showed the opposite relationship, with large total stem area and small total stem diameter. Triploids maintained large stem area and large stem diameter across all three years. Triploids also had the greatest specific gravity across all three years. Overall, regardless of genotype or ploidy, specific gravity increased from year one to year two and then dropped in year three.

**Table 1:** ANOVA results for traits measured across the three years of growth

			**Source effect *p***-**value**		
**Trait**	**Year**	**Block**	**Genotype**	Genotype × Block*	**Year × Genotype**
Height	<0.0001	<0.0001	<0.0001	<0.0001	<0.0001
Stem Diameter	<0.0001	<0.0001	<0.0001	<0.0001	0.4603
Stem Area	<0.0001	<0.0001	<0.0001	0.0480	0.0939
Stem Number	0.0010	<0.0001	<0.0001	<0.0001	0.0006
Specific Gravity	<0.0001	0.2683	<0.0001	0.9295	0.2970
	**Year**	**Block**	**Ploidy**	**Ploidy × Block**	**Year × Ploidy**
Height	<0.0001	0.0009	<0.0001	0.0022	0.0537
Stem Diameter	<0.0001	<0.0001	<0.0001	0.8771	0.8960
Stem Area	<0.0001	<0.0001	<0.0001	0.0207	0.0005
Stem Number	<0.0001	0.0006	<0.0001	0.9446	0.6596
Specific Gravity	<0.0001	0.6811	<0.0001	0.3008	0.6419

*Height, stem diameter, stem area, and stem number are the highest in block one, block three is the lowest.

Height, total stem diameter, and total stem area strongly correlated with yield over all three years of growth (Table 2; Additional file 3). There was a weak correlation between specific gravity and yield across all three years, however, when the genotypes were separated by ploidy, only the diploids had a correlation between specific gravity and yield in years two and three. Stem number did not correlate with yield for any year of growth, except when the data were analyzed with regard to ploidy. Stem number correlated with mean yield for diploids and triploids in the first year of growth and only triploids in the second year of growth. Height and total stem area displayed the strongest correlations with yield than any of the other traits.

**Table 2:** Correlations of yearly measurements with third year yields

	**Stem diameter**	**Stem area**	**Stem number**	**Specific gravity**	**Yield**
**1**^**st **^**year post**-**coppice**					
Height	0.08494	**0.52578**	−**0.25028**	0.08061	**0.67611**
Stem Diameter	-	**0.71701**	**0.85927**	−0.09223	**0.28958**
Stem Area		-	**0.28000**	−0.07587	**0.63675**
Stem Number			-	−0.07622	−0.02927
Specific Gravity				-	**0.13362**
**2**^**nd **^**year post**-**coppice**					
Height	0.04699	**0.48335**	−0.27203	**0.19114**	**0.78023**
Stem Diameter	-	**0.72570**	**0.86908**	−0.10570	**0.28428**
Stem Area		-	**0.31578**	−0.5500	**0.61371**
Stem Number			-	−**0.13163**	−0.03442
Specific Gravity				-	**0.23658**
**3**^**rd **^**year post coppice**					
Height	0.04407	**0.57593**	−**0.27513**	0.11805	**0.77364**
Stem Diameter	-	**0.55106**	**0.39146**	0.04623	**0.31534**
Stem Area		-	0.02388	−0.01120	**0.70359**
Stem Number			-	−0.01989	−0.11433
Specific Gravity				-	**0.13155**

All significant correlations at α ≤ 0.05 are highlighted in bold.

### Allometric modeling of yield

**Figure 5:**
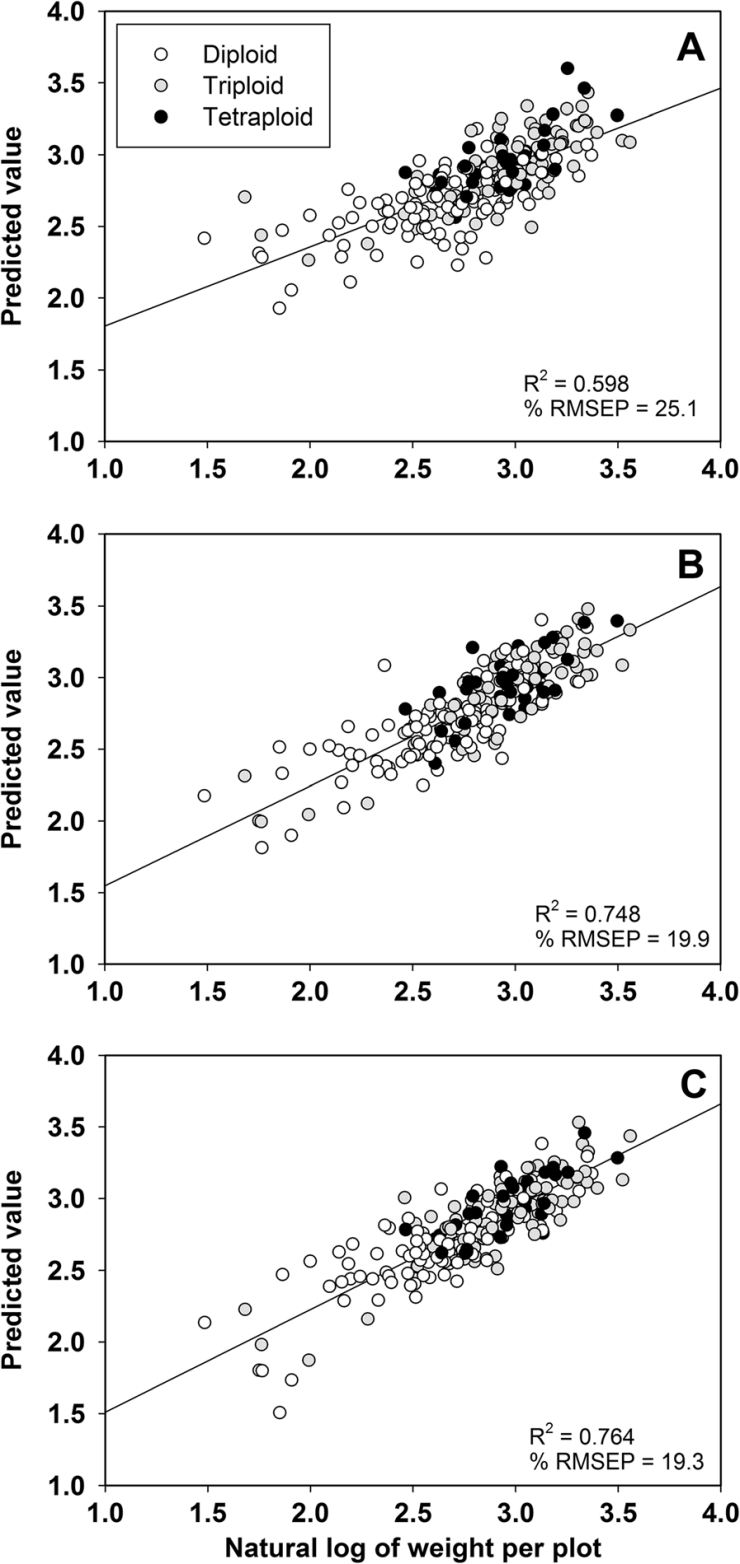
**Ordinary least-squared regression models for estimating third year biomass yield from yearly measurements A) First year post**-**coppice, B) Second year post-coppice, C) Third year post-coppice.** Each data point represents one genotype shaded by ploidy.

Height, total stem area, and specific gravity were used as variables in allometric models to identify relationships between yield and the yearly growth measurements. Total stem diameter was found to be an insignificant variable in the models and was removed. Multiple linear regression models used to estimate third-year harvest yield were significant for all three years of growth (Table 3; Figure 5). Among third-year measurements, specific gravity was not a significant predictor in the model. Height was the strongest predictor of yield across all three years.

**Table 3:** **Parameter estimates and p**-**values for ordinary least**-**squares regressions for all 75 genotypes each year of growth**

		**Coefficients**			**P**-**value**		
**Data set**	***a***	***b***	***c***	***d***	**Height**	**Area**	**Specific gravity**
1^st^ year post-coppice	−6.425	1.111	0.554	1.013	<0.0001	<0.0001	0.007
2^nd^ year post-coppice	−12.701	2.083	0.430	1.332	<0.0001	<0.0001	<0.0001
3^rd^ year post-coppice	−10.670	1.659	0.544	0.560	<0.0001	<0.0001	0.137

*a* is a constant; *b*, *c*, and *d* are slopes of the parameters height, area, and diameter, respectively.

## Discussion

Polyploidy is a common phenomenon among species of willow, and may have been a strong contributor to speciation through reproductive isolation [18]. Many species of willow hybridize in nature and even more can be forced to hybridize through controlled pollination, including those with different ploidy. *Salix miyabeana*, native to Japan, Korea, and China, is a tetraploid species [19]. This species has been exploited in recent breeding of willow to produce triploid progeny with superior performance, as was observed in this study and in the work of others (I. Shield, personal communication) [5]. Based on previous breeding and yield analysis that has led to the development of commercial cultivars such as ‘Tully Champion’, ‘Fabius’, ‘Millbrook’, and ‘Oneida’, it was expected that hybrid progeny with pedigrees *S. viminalis* × *S. miyabeana* and *S. purpurea* × *S. miyabeana* would likely perform well and produce high yields [1,2]. However, the results from this selection trial with 75 genotypes representing various ploidy levels suggest that the basis for this improved performance is associated with the triploid nature of these hybrids. It would be expected that a diploid by tetraploid cross would produce a sterile triploid assuming normal segregation of gametes during meiosis; however progeny have been recovered from crosses using a triploid as the male parent in a controlled pollination of a diploid mother. These progeny were likely the products of an abnormal meiotic division resulting in unreduced gametes in the form of pollen with an even number of chromosomes - in one case producing a triploid, *S. viminalis* × (*S. viminalis* × *S. miyabeana*) ‘Sheridan’ and in the other case, producing the two tetraploid individuals in the trial, *S. koriyanagi* × (*S. purpurea* × *S. miyabeana*) 01X-264-024 and 01X-264-033, siblings produced from a cross of a diploid by a triploid.

### Heterosis and ploidy

The selection trial established and analyzed is this study consisted of a comprehensive collection of new progeny produced from controlled crosses performed in 2001, 2002, and 2005, many of which involved *S. miyabeana* as a parent. Within the trial many of the parents were included along with the selected progeny for the purpose of examining heterosis, as well as any transgressive segregation, often observed among hybrid progeny in willow and other highly heterozygous species. In this trial, the majority of the high yielding genotypes were triploid progeny of a naturally tetraploid *S. miyabeana* parents, whereas many of the diploid progeny were lower yielding. This was clearly demonstrated for several of the families in the trial. The 05X-281 triploid progeny and the 05X-276 diploid progeny are half-sibs with the same diploid mother, *S. koriyanagi* × *S. purpurea* ‘Allegany’. The 05X-281 triploid progeny were among the top yielding, with (*S. koriyanagi* × *S. purpurea*) × *S. miyabeana* 05X-281-068 producing the greatest yield in the trial and outperforming its diploid mother, *S. koriyanagi* × *S. purpurea* ‘Allegany’ and its tetraploid father, *S. miyabeana* ‘SX67’. In contrast, nearly all of the 05X-276 diploid progeny all produced smaller yields than either their diploid mother or their diploid father, *S. suchowensis* P63 (only one had slightly higher yield than ‘Allegany’). In effect, only the 05X-281 triploid progeny displayed heterosis, not the half-sib diploid progeny. This was also observed among the 05X-293 and the 05X-295 progeny, half-sibs with *S. purpurea* 05-01-002 as the common maternal parent. The triploid 05X-295 progeny displayed heterosis with all progeny greater than the mid-parent mean, with two having greater yield than both parents. Only three of the diploid 05X-293 progeny were included in this study. Two were only slightly higher in yield than 05-01-002, and the third had significantly greater yield than both parents.

The performance of diploid vs. triploid half-sib progeny suggests that in willow, heterosis is not directly a result of heterozygosity at deleterious loci, since both types of progeny are likely to be highly heterozygous based on previous characterization of microsatellites in willow. A better explanation or a more likely explanation for heterosis observed in polyploids may be gene dosage effect. This has recently been observed in triploid maize [20] building on the proposed hypothesis by Birchler and Veitia [21] that heterosis in polyploid individuals is similar to quantitative traits controlled by genes with additive effects. In addition, it is becoming clear that heterosis in polyploids and hybrids is largely due to complex molecular changes at epigenetic, genomic, proteomic, and metabolic levels [10,22,23]. Gene expression changes in response to the previously mentioned biological changes have been shown to have significant impact on stress responses and phytohormone signaling in allotetraploids of *A. thaliana*[24]. We are presently using transcriptomics to compare patterns of gene expression in triploid willows to those in diploids and tetraploids to test if expression of critical genes is correlated with gene dosage.

### Biomass growth traits

While this particular study does not address the underlying molecular differences between genotypes with different ploidy, we did observe key differences in growth patterns correlated with ploidy. These differences by ploidy were significant despite the high level of variation among all the genotypes. While stem number may not have a significant correlation with yield, stem number did vary significantly by ploidy (Tables 1 and 2). Diploids have the largest number of stems per plant, resulting in large total stem diameter, but the lowest total stem area (Figure 4). Tetraploids had the lowest number of stems per plant, resulting in the lowest total stem diameter, but high total stem area. While tetraploids had large total stem area and tall stem height, they had low specific gravity, reducing their overall biomass yield. Tetraploids grew fast, in terms of diameter and volume, but the low specific gravity in the wood reduced their potential biomass yield. Triploids, on the other hand, produced stem numbers intermediate between those of diploids and tetraploids, with the greatest total stem area and the greatest specific gravity, putting them at the top with regard to yield. Triploid aspen were also identified as having greater growth rates and greater specific gravity than diploids [25-27]. Previous work examining morphological traits in willow showed similar results with regard to diploids and tetraploids, however no triploids were ever examined [28]. The tetraploids examined in Tharaken et al. [28] Previous work examining morphological traits in willow showed similar results with regard to diploids and tetraploids, however no triploids were ever examined [28]. The tetraploids examined in Tharaken et al. [28] clustered separately from all the diploids and were characterized by a small number of large diameter stems and low specific gravity, similar to what was observed in our trial.

Our analysis of first-, second-, and third-year stems revealed that specific gravity does not continue to increase with age, similar to what has been seen in poplar [29]. Differences in specific gravity along the length of the stem have been previously observed in willow and in poplar, making sampling at the same location on the stem critical for comparisons. For shrub willow, pith can form in the third year of growth and may explain the drop in specific gravity measured in the third-year stems, but more detailed anatomical studies are needed to verify this. Negative relationships between specific gravity and volume growth rates have been observed among populations of poplar [30,31], however in this study no clear relationship between volume measurements and specific gravity could be identified. There were minor negative relationships observed among triploids in their first year between specific gravity and stem number, total stem area and total stem diameter, but there was a positive relationship between specific gravity and stem number for diploids in the same year. Among diploids in their second and third years of growth, there was a positive relationship between height and specific gravity. Across the entire trial, there was a correlation between specific gravity and yield, but it was weak.

Among all of the traits analyzed in this study, the strongest correlation with yield was observed for height and total stem area across all three years. Tharakan et al. [28] also identified a strong and significant correlation between height and biomass yield. Total stem diameter was strongly correlated with total stem area and stem number. Across all genotypes, regardless of ploidy, and across all years, total stem area displayed a stronger correlation with yield than total stem diameter. Since there are many stems produced by each plant, total stem area better reflects aboveground volume than total stem diameter. In optimizing allometric equations to estimate yield, height and total stem area were critical parameters, while total stem diameter was an insignificant variable.

### Estimating yield from allometric regressions

Allometric regressions have been used in the forestry community for decades to predict biomass production from volume measurements, mainly diameter-at-breast-height (DBH) and height [32-34]. Typically, stem diameter measurements for shrub willow have been collected at the lowest practical point in the stem to take the measurement with digital calipers, approximately 20 to 30 cm above the soil surface. These numbers are then used to calculate total stem area, which is somewhat analogous to the basal area measurement of trees. Using models that rely on height, total stem area, and specific gravity, it is possible to estimate third year biomass yield with 60% accuracy. Third year measurements estimated third year yield the best with an R^2^ of 0.76. These models clearly do not address all of the variation associated with aboveground biomass production in shrub willow. One variable that is unaccounted for in these models is branching, a trait that contributes to the final biomass yield, but is not easily measured. Arevalo et al. [35] was able to estimate aboveground biomass from four willow genotypes using diameter-based allometric equations. Height was not found to be significant, which contradicts the findings in the current study. Model development in Arevalo et al. [35] was based off of single stems cut and dried, not entire plants, which would reduce the amount of variation within the model. More research needs to be done to understand the relationship between whole plant growth and final yield in shrub willow.

## Conclusions

We have demonstrated that triploid shrub willow produce higher yield than their diploid and tetraploid parents. The mean yield of the top five performing new genotypes (all triploid) in this trial was 16 oven dry Mg ha^−1^ yr^−1^, while the mean yield of the top five current commercial cultivars was 14 oven dry Mg ha^−1^ yr^−1^. This represents an improvement of 12.5% in yield over current commercial cultivars on this single site. Larger scale yield and evaluation trails with a selection of these top genotypes have been planted in NY, PA, and WV to further test their performance prior to final selection, scale-up, and commercial deployment. Overall the three classes of genotypes based on ploidy had significantly different growth patterns and specific gravity of the stem biomass. Strong correlations were identified among all of these traits. Correlations with yield indicate that there are many factors and their interaction impacting willow biomass production. Understanding how first and second year growth measurements, such as total stem area and height, relate to final yield and how these traits change over a three-year rotation period will allow for the possible selection and prediction of performance of genotypes within the first year of growth.

## Methods

### Selection trial establishment, measurement, and harvest

The trial was established in Geneva, NY at Cornell University’s New York State Agricultural Experiment Station (42°52’48” N, 77°00’55” W). The soil at the Geneva, NY site is a poorly drained Odessa silt loam with a depth to water table of 25 to 45 cm [36,37]. Soil samples were collected to a depth of 25 cm with a 2-cm diameter soil probe (AMS, American Falls, ID) from eight random locations in a block, pooled, and homogenized. This was repeated for each of the three blocks and the sample for each block was analyzed by the Cornell Nutrition Analysis Laboratory (Ithaca, NY). Mean values were calculated for the three samples (Table 4). Precipitation records were obtained from the Northeast Regional Climate Center from a weather station in Geneva, NY.

**Table 4:** **Site characteristics for Geneva**, **NY**

**Site characteristics**	**Geneva**, **NY**
Latitude	42°52’48” N
Longitude	77°00’55” W
Elevation (m)	185
Soil Type	Odessa silt loam
2008 Precipitation (May – August; cm)	35.5
2009 Precipitation (May – August; cm)	30.3
2010 Precipitation (May – August; cm)	47.9
2011 Precipitation (May – August; cm)	35.8
Phosphorus (mg kg^−1^)	1.53 ± 0.50
Potassium (mg kg^−1^)	64.7 ± 0.12
Magnesium (mg kg^−1^)	336 ± 11.6
Calcium (mg kg^−1^)	2676 ± 105
Iron (mg kg^−1^)	1.63 ± 0.40
Aluminum (mg kg^−1^)	11.63 ± 2.61
Manganese (mg kg^−1^)	8.87 ± 0.71
Zinc (mg kg^−1^)	0.53 ± 0.21
Nitrate (mg kg^−1^)	3.67 ± 3.2
pH	6.45 ± 016
Buffer pH	6.07 ± 0.20
% Organic Matter	5.20 ± 0.31

The site, which was previously in perennial grass cover, was prepared by killing existing vegetation with glyphosate, followed by moldboard plowing and disking. Rooted plants were established from 10-cm cuttings in potting mix in tubes in a greenhouse, which were transplanted to the field in May 2008 in double row spacing (0.61 m between plants within a row, 0.76 m between rows, with 1.52 m alleys between double rows, resulting in a planting density of 15,000 plants ha^−1^. Each genotype was planted in 24-plant plots with six plants per row (Additional file 1). The four rows included one-half of a double row, a complete double-row, and half of a double-row, such that the middle two rows were in a double-row bordered on either side by a single-row of the same genotype. The middle eight plants in the center double row were designated as the measurement plot. Both sides of the field were planted with a single row of ‘Fish Creek’ from unrooted cuttings to complete those double-rows and single cuttings of ‘Fish Creek’ were planted on each end of every row to provide a border around the plots. The trial was a randomized complete block design with three replicate blocks. During the establishment year, a combination of hand-weeding and spot herbicide (glyphosate) application was used for weed management. In December 2008 after the establishment year growing season, survival was surveyed and the plants were coppiced with a brush saw. Following each growing season, measurements of stem growth were collected. Height of the tallest stem was measured with a (Crain Enterprises, Inc, Mound City, IL) height pole for each of the four plants at the center of each plot, and the mean was calculated for each plot. Stem number per plant was counted and the diameter of every stem 5 mm or larger was measured using a Racal 500 computerized caliper (Masser, Rovaniemi, Finland) for each plant within the measurement plot. Stem diameters were measured at least 20 cm, but not higher than 30 cm above the soil surface. The stem diameters were used to calculate total stem diameter per plant and total stem area per plant, and then means for those values were calculated for the eight plants in each plot. In addition, a single stem from a border row plant was selected and a 25-cm section was collected from the middle of a typical canopy stem of one plant in each plot to determine specific gravity by volumetric displacement [38]. After the third season post-coppice in December 2011, aboveground biomass was harvested from each eight-plant measurement plot using a Ny Vraa JF192 harvester and fresh biomass was weighed as chips in a plastic fruit bin mounted on three Avery Weigh-Tronix weigh cells, each with a 900 kg capacity (item # 53412–0019, Fairmont, MN) A sub-sample of chips was collected from each plot, dried to constant weight at 65°C, and weighed to determine moisture content at harvest, which was then used to derive plot dry weights from the fresh weight measurements. Yields were calculated based on the area of the eight-plant measurement plot and annual yields were calculated by dividing the dry biomass yield at harvest by three.

### Flow cytometry

Analysis of 50 mg of mature leaf tissue from parental genotypes and selected progeny was performed at the Flow Cytometry and Imaging Core Laboratory at Virginia Mason Research Center in Seattle, WA. Each batch of samples submitted included a diploid and tetraploid check to account for variation in instrumental error. Samples were prepared and analyzed using their standard protocol [39]. Suspensions of sample nuclei was spiked with suspension of standard nuclei (chicken erythrocyte nuclei (CEN), 2.5 pg 2C^−1^) and analyzed with a FACS Calibur flow cytometer (Becton-Dickinson, San Jose, CA). For each measurement, the propidium iodide fluorescence area signals from 1,000 nuclei were collected and analyzed by CellQuest software (Becton-Dickinson, San Jose, CA). The mean position of the G0/G1 nuclei peak of the sample and the internal standard were determined by CellQuest software. The mean nuclear DNA content of each plant sample, measured in pg, was based on 1,000 scanned nuclei.

To account for day to day variability and instrumental error, all ploidy data was standardized to bring all of the variables into proportion with one another for comparison. A diploid check clone *S. purpurea* 94006 was included for most batches of samples ran at the same time on the flow cytometer. However, when 94006 was not included, a similar diploid check *S. purpurea* 94001 was used to standardize values. All values for the 94006 check from multiple runs were averaged and then divided by the value of the check for that run. This factor was then multiplied by each of the sample values within the same batch as the check and is reported in Additional file 1. When a genotype was analyzed more than once, the pg 2C^−1^ values were averaged.

### Statistical analysis

All statistical analyses were performed using SAS® version 9.3 at a critical *α* level of 0.05 [40]. Normality of distribution for each trait was checked by Kolmogorov D and Shapiro-Wilk’s statistic W and graphically by normal-probability plots and histograms using SAS PROC UNIVARIATE. No transformation of the data was required. PROC GLM was used to perform analysis of variance and to estimate significant effects of the factors and any interactions. Analysis of genotype and ploidy were kept independent of each other because combining both genotype and ploidy into the same model led to nesting of genotype within ploidy, preventing comparison of all genotypes to each other. PROC CORR was used to identify any significant correlations among variables obtained in this study.

Data sets from annual measurements were used to estimate model parameters for the allometric relationship between total stem area, height, specific gravity and biomass yield. A multiple linear regression with logarithmic transformation of both dependent (except for specific gravity) and independent variables was performed using PROC REG. The regression model was:

$$\ln\left(W\right)=a+b\ln\left(H\right)+c\ln\left(A\right)+d\left(D\right)$$

where W is the dry weight per plot (kg), *H* is the mean height per plot (cm), *A* is the total stem area per plot (cm^2^), *D* is the mean wood specific gravity (g/cm^3^), *a* is a constant, and *b*, *c* and *d* are slopes of the parameters.

## Competing interests

LBS is an inventor on plant patents for ‘Fish Creek’, ‘Millbrook’, and ‘Owasco’ and receives royalty payments from the sale of those and other willow cultivars included in this paper. LBS will be listed as an inventor on future plant patent applications that may be filed on certain genotypes described in this paper. The other authors have no competing interests.

## Authors’ contributions

MJS carried out analysis of the data and drafted the manuscript. FEG conducted ploidy analysis and collected and analyzed growth measurement data. LBS conceived of the study, contributed to trial establishment, maintenance, and harvesting, and provided overall project coordination. All authors read and approved the final manuscript.

## Authors’ information

MJS current affiliation and contact information: Eastern Regional Research Center Sustainable Biofuels and Coproducts Research Unit 600 East Mermaid Lane Wyndmoor, PA 19038 michelle.serapiglia@ars.usda.gov.

## Supplementary Material

Additional file 1 **PDF format.** List of genotypes studied, their pedigrees, and their ploidy as determined by flow cytometry. Click here for file

Additional file 2 **PDF format.** Harvested biomass per plot at three years post-coppice. Bars represent mean ± standard error. Bars are shaded by ploidy of the genotype. Letters indicate results of means separation test. Click here for file

Additional file 3 **PDF format.** Correlations between traits, calculated by age and ploidy. Click here for file
