# Supplementary material for: Early selection of novel triploid hybrids of shrub willow with improved biomass yield relative to diploids
Source: BMC Plant Biol. 2014 Mar 24;14:74. doi: 10.1186/1471-2229-14-74 (PMC3987697; doi:10.1186/1471-2229-14-74)
Supplement: Additional file 1 — PDF format. List of genotypes studied, their pedigrees, and their ploidy as determined by flow cytometry. [file 1471-2229-14-74-S1.pdf]

Clone ID/Cultivar Epithet	Species/Pedigree	Nuclear DNA content (pg/2C)	Estimated Ploidy
'SV1'	*S.* × *dasyclados*	0.87	2X
'S25'	*S. eriocephala*	0.90	2X
01-07-252	*S. eriocephala*	1.16	2X
05X-298-003	*S. eriocephala* (01-03-210) × *S. eriocephala* (9837-004)	1.17	2X
05X-298-019	*S. eriocephala* (01-03-210) × *S. eriocephala* (9837-004)	-	2X
05X-298-035	*S. eriocephala* (01-03-210) × *S. eriocephala* (9837-004)	-	2X
05X-298-038	*S. eriocephala* (01-03-210) × *S. eriocephala* (9837-004)	-	2X
05X-299-016	*S. eriocephala* (01-03-210) × *S. eriocephala* (01-06-085)	-	2X
05X-299-044	*S. eriocephala* (01-03-210) × *S. eriocephala* (01-06-085)	1.02	2X
05X-299-045	*S. eriocephala* (01-03-210) × *S. eriocephala* (01-06-085)	-	2X
P63	*S. suchowensis*	0.86	2X
05X-278-005	*S. integra* (P336) × *S. suchowensis* (P63)	0.77	2X
05X-278-016	*S. integra* (P336) × *S. suchowensis* (P63)	-	2X
05X-278-057	*S. integra* (P336) × *S. suchowensis* (P63)	-	2X
05X-278-071	*S. integra* (P336) × *S. suchowensis* (P63)	-	2X
05X-278-073	*S. integra* (P336) × *S. suchowensis* (P63)	-	2X
'Boonville'	*S. koriyanagi* (SH3) × *S. purpurea* (94001)	1.04	2X
01X-265-019	*S. koriyanagi* (SH3) × *S. purpurea* (94001)	-	2X
01X-265-027	*S. koriyanagi* (SH3) × *S. purpurea* (94001)	-	2X
'Onondaga'	*S. koriyanagi* (SH3) × *S. purpurea* (94002)	0.94	2X
00-01-088	*S. purpurea*	1.07	2X
01-01-032	*S. purpurea*	1.09	2X
05-01-002	*S. purpurea*	1.01	2X
95026	*S. purpurea*	0.90	2X
05X-279-074	*S. purpurea* (94006) × *S. purpurea* (02-201-005)	1.04	2X
05X-279-080	*S. purpurea* (94006) × *S. purpurea* (02-201-005)	-	2X
'Fish Creek'	*S. purpurea* (94006) × *S. purpurea* (94001)	1.02	2X
05X-293-021	*S. purpurea* (05-01-002) × *S. purpurea* 'Fish Creek'	-	2X
05X-293-047	*S. purpurea* (05-01-002) × *S. purpurea* 'Fish Creek'	-	2X

05X-293-053	*S. purpurea* (05-01-002) × *S. purpurea* 'Fish Creek'	1.06	2X
'Allegany'	*S. koriyanagi* (SH3) × *S. purpurea* (95058)	0.95	2X
05X-297-028	(*S. koriyanagi* × *S. purpurea*) 'Allegany' × *S. purpurea* (99239-011)	1.12	2X
05X-276-057	(*S. koriyanagi* × *S. purpurea*) 'Allegany' × *S. suchowensis* (P63)	1.12	2X
05X-276-061	(*S. koriyanagi* × *S. purpurea*) 'Allegany' × *S. suchowensis* (P63)	-	2X
05X-276-065	(*S. koriyanagi* × *S. purpurea*) 'Allegany' × *S. suchowensis* (P63)	-	2X
05X-276-076	(*S. koriyanagi* × *S. purpurea*) 'Allegany' × *S. suchowensis* (P63)	-	2X
05X-275-024	*S. purpurea* (01-01-032) × *S. suchowensis* (P63)	1.15	2X
05X-275-026	*S. purpurea* (01-01-032) × *S. suchowensis* (P63)	-	2X
02X-326-010	*S. miyabeana* (9970-021) × (*S. viminalis* × (*S. schwerinii* × *S. viminalis*)) 'Olof'	-	3X
02X-326-015	*S. miyabeana* (9970-021) × (*S. viminalis* × (*S. schwerinii* × *S. viminalis*)) 'Olof'	1.15	3X
05X-295-011	*S. purpurea* (05-01-002) × *S. miyabeana* 'SX64'	1.21	3X
05X-295-013	*S. purpurea* (05-01-002) × *S. miyabeana* 'SX64'	-	3X
05X-295-014	*S. purpurea* (05-01-002) × *S. miyabeana* 'SX64'	-	3X
05X-295-015	*S. purpurea* (05-01-002) × *S. miyabeana* 'SX64'	-	3X
05X-295-020	*S. purpurea* (05-01-002) × *S. miyabeana* 'SX64'	1.21	3X
'Millbrook'	*S. purpurea* (95026) × *S. miyabeana* 'SX64'	1.24	3X
05X-292-035	*S. purpurea* (95026) × *S. miyabeana* (01-200-003)	1.24	3X
05X-292-042	*S. purpurea* (95026) × *S. miyabeana* (01-200-003)		3X
05X-281-034	(*S. koriyanagi* × *S. purpurea*) 'Allegany' × *S. miyabeana* 'SX67'	-	3X
05X-281-043	(*S. koriyanagi* × *S. purpurea*) 'Allegany' × *S. miyabeana* 'SX67'	1.21	3X
05X-281-051	(*S. koriyanagi* × *S. purpurea*) 'Allegany' × *S. miyabeana* 'SX67'	-	3X
05X-281-060	(*S. koriyanagi* × *S. purpurea*) 'Allegany' × *S. miyabeana* 'SX67'	-	3X
05X-281-064	(*S. koriyanagi* × *S. purpurea*) 'Allegany' × *S. miyabeana* 'SX67'	-	3X
05X-281-066	(*S. koriyanagi* × *S. purpurea*) 'Allegany' × *S. miyabeana* 'SX67'	-	3X
05X-281-068	(*S. koriyanagi* × *S. purpurea*) 'Allegany' × *S. miyabeana* 'SX67'	1.28	3X
05X-281-071	(*S. koriyanagi* × *S. purpurea*) 'Allegany' × *S. miyabeana* 'SX67'	1.29	3X
05X-291-006	*S. purpurea* (00-01-088) × *S. miyabeana* 'SX67'	-	3X

05X-291-021	*S. purpurea* (00-01-088) × *S. miyabeana* 'SX67'	-	3X
05X-291-049	*S. purpurea* (00-01-088) × *S. miyabeana* 'SX67'	1.26	3X
05X-291-050	*S. purpurea* (00-01-088) × *S. miyabeana* 'SX67'	1.22	3X
'Sheridan'	*S. viminalis* (SV2) × (*S. viminalis* × *S. miyabeana*) (99207-019)	1.22	3X
'Owasco'	*S. viminalis* (SV7) × *S. miyabeana* 'SX64'	1.21	3X
01X-271-009	*S. viminalis* (SV7) × *S. miyabeana* 'Canastota'	1.15	3X
02X-324-001	((*S. schwerinii* × *S. viminalis*) × *S. viminalis*) 'Torhild' × *S. miyabeana* 'SX64'	1.20	3X
01-200-001	*S. miyabeana*	1.48	4X
'SX61'	*S. miyabeana*	1.53	4X
'SX64'	*S. miyabeana*	1.55	4X
'SX67'	*S. miyabeana*	1.52	4X
05X-287-013	*S. miyabeana* (01-200-001) × *S. miyabeana* 'SX64'	1.54	4X
05X-287-033	*S. miyabeana* (01-200-001) × *S. miyabeana* 'SX64'	-	4X
05X-287-089	*S. miyabeana* (01-200-001) × *S. miyabeana* 'SX64'	-	4X
05X-287-096	*S. miyabeana* (01-200-001) × *S. miyabeana* 'SX64'	-	4X
'Sherburne'	*S. miyabeana* 'SX61' × *S. miyabeana* 'SX67'	1.73	4X
01X-264-024	*S. koriyanagi* (SH3) × (*S. purpurea* × *S. miyabeana*) (99218-013)	1.85	4X
01X-264-033	*S. koriyanagi* (SH3) × (*S. purpurea* × *S. miyabeana*) (99218-013)	1.82	4X
