# Supplementary figures and images for: Early selection of novel triploid hybrids of shrub willow with improved biomass yield relative to diploids
Source: BMC Plant Biol. 2014 Mar 24;14:74. doi: 10.1186/1471-2229-14-74 (PMC3987697; doi:10.1186/1471-2229-14-74)

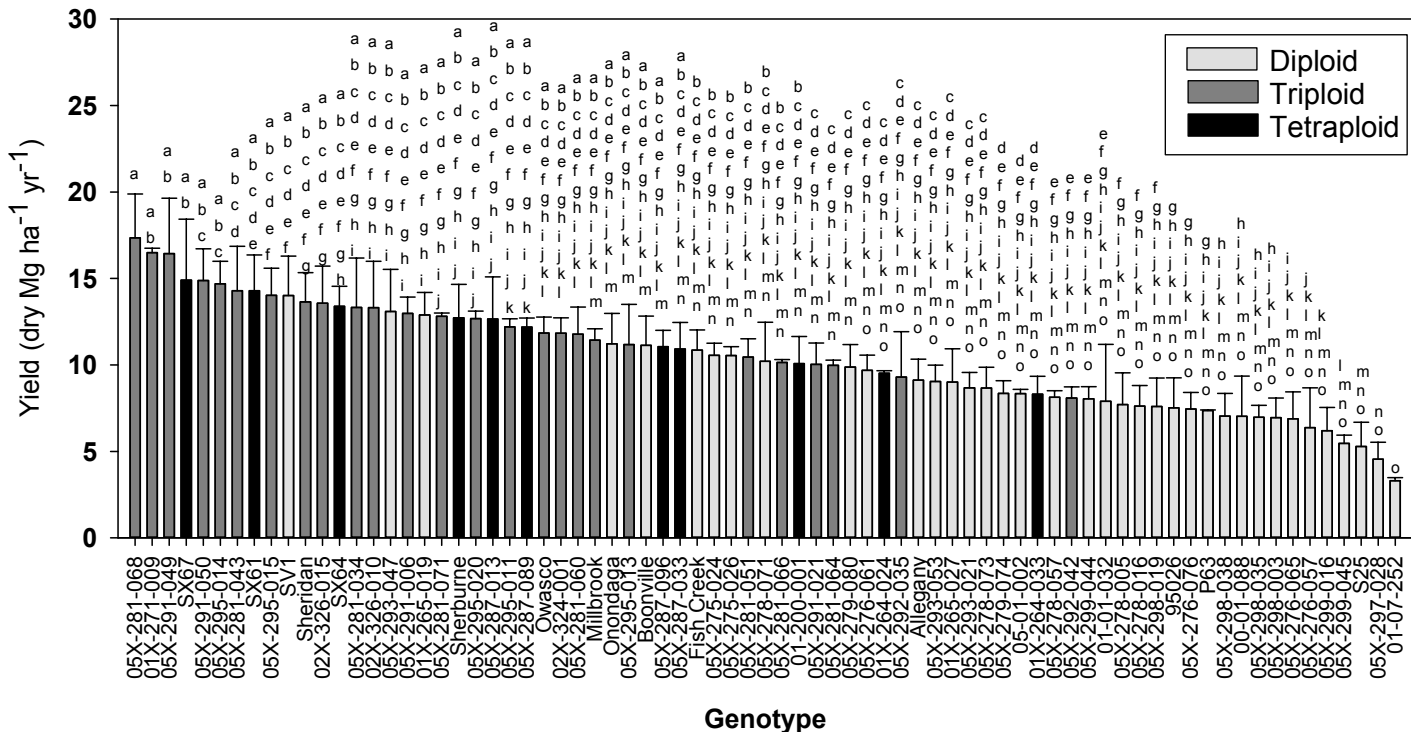

Supplement: Additional file 2 — PDF format. Harvested biomass per plot at three years post-coppice. Bars represent mean ± standard error. Bars are shaded by ploidy of the genotype. Letters indicate results of means separation test. [file 1471-2229-14-74-S2.pdf]
