# Supplementary material for: Early selection of novel triploid hybrids of shrub willow with improved biomass yield relative to diploids
Source: BMC Plant Biol. 2014 Mar 24;14:74. doi: 10.1186/1471-2229-14-74 (PMC3987697; doi:10.1186/1471-2229-14-74)
Supplement: Additional file 3 — PDF format. Correlations between traits, calculated by age and ploidy. [file 1471-2229-14-74-S3.pdf]

	Stem Diameter	Stem Area	Stem Number	Specific Gravity	Yield
**1st year Diploids**					
Height	0.15216	**0.41634**	-0.09562	0.08700	**0.59889**
Stem Diameter	-	**0.80831**	**0.88835**	-0.10531	**0.36281**
Stem Area		**-**	**0.45810**	-0.10142	**0.47663**
Stem Number			-	-0.08083	**0.20277**
Specific Gravity				-	0.16692
**1st year Triploids**					
Height	0.10276	**0.40136**	-0.17533	0.09545	**0.48699**
Stem Diameter	-	**0.85038**	**0.90007**	**-0.28490**	**0.47751**
Stem Area		-	**0.55154**	**-0.23157**	**0.62834**
Stem Number			-	**-0.28530**	**0.24399**
Specific Gravity				-	-0.01739
**1st year Tetraploids**					
Height	0.27339	**0.46548**	0.12416	-0.00272	**0.62244**
Stem Diameter	-	**0.69956**	**0.90635**	0.30457	0.19188
Stem Area		-	**0.34992**	0.06816	**0.54519**
Stem Number			-	**0.38477**	-0.03170
Specific Gravity				-	0.09185
**2nd year Diploids**					
Height	0.07422	**0.35499**	-0.14456	**0.39886**	**0.74136**
Stem Diameter	-	**0.80934**	**0.90566**	-0.16670	**0.33480**
Stem Area		-	**0.49844**	-0.00212	**0.46514**
Stem Number			-	**-0.24240**	0.15519
Specific Gravity				-	**0.42257**
**2nd year Triploids**					
Height	0.00022	**0.22789**	-0.21149	0.12006	**0.59223**
Stem Diameter	-	**0.84703**	**0.88579**	-0.10949	**0.42434**
Stem Area		-	**0.53668**	-0.10175	**0.52910**
Stem Number			-	-0.07484	**0.22180**
Specific Gravity				-	0.15289
**2nd year Tetraploids**					
Height	0.36713	**0.62027**	0.03881	**-0.34238**	**0.54643**
Stem Diameter	-	**0.78727**	**0.84581**	-0.03484	0.28539
Stem Area		-	**0.35178**	-0.16115	**0.60179**
Stem Number			-	0.05084	-0.11141
Specific Gravity				-	-0.02115
**3rd year Diploids**					
Height	0.12598	**0.47482**	**-0.18728**	**0.33069**	**0.72209**
Stem Diameter	**-**	**0.70245**	**0.39833**	0.02770	**0.45438**
Stem Area		-	**0.18781**	0.13603	**0.63476**
Stem Number			-	-0.10155	0.04654
Specific Gravity				-	**0.31078**
**3rd year Triploids**					
Height	0.03447	**0.35545**	-0.12944	0.02758	**0.58145**

Stem Diameter	**-**	**0.65617**	**0.23312**	-0.18129	**0.43821**
Stem Area	**-**	-	0.13998	-0.15099	**0.50511**
Stem Number			-	-0.17453	0.02739
Specific Gravity				-	0.11502
**3rd year Tetraploids**					
Height	0.03195	**0.41022**	-0.22185	-0.06590	**0.67332**
Stem Diameter	**-**	**0.64895**	**0.77743**	0.14234	0.27458
Stem Area		-	0.07488	-0.03822	**0.67847**
Stem Number			-	0.16883	-0.13934
Specific Gravity				-	-0.00247
